# A Simplified Technique for Arthroscopic Reduction and Double-Pulley Fixation of Bony Bankart Lesion by Percutaneous Spinal Needle Suture

**DOI:** 10.1016/j.eats.2025.103508

**Published:** 2025-03-18

**Authors:** Fashuai Wu, Wenbo Yang, Chunqing Meng, Hong Wang, Wei Huang

**Affiliations:** Department of Orthopaedics, Union Hospital, Tongji Medical College, Huazhong University of Science and Technology, Wuhan, China

## Abstract

Bony Bankart lesion is commonly seen and highly associated with anterior glenohumeral instability. However, the reported arthroscopic techniques that reduce and fix bony Bankart lesion with traditional suture devices may encounter several problems during the suture-passing process, including splitting the fracture fragment or surrounding soft tissue, resulting in iatrogenic damage, and causing difficulties for instrument operation and suture management. Here, we have proposed a simplified technique for arthroscopic reduction and fixation of bony Bankart lesion by the introduction of percutaneous suturing with a spinal needle, which has a lower risk of iatrogenic damage, lower possibility of splitting the fracture fragment or surrounding soft tissue, and less difficulty in suturing during operation and suture management. Our technique, a combination of percutaneous spinal needle suture passing and s double-pulley suture-tying technique, is less invasive to patients, more convenient and economical, and more suitable for beginners, which is challenging to achieve with a traditional suture device. In addition, the equipment used in our technique is commonly seen and accessible in the operating room, without requiring high-value consumables and supporting instruments. In summary, our technique, integrating economy, simplicity, reliability, and safety, has very good application potential in clinical situations.

Glenoid fractures mostly occur in young patients at an average age of 35 years under 2 clinical scenarios: high-energy trauma and instability events.^1^ Intra-articular glenoid fractures can be classified into 6 categories according to the most widely used Ideberg-Goss classification system; bony Bankart lesion, an avulsion of the glenohumeral labral complex associated with anterior glenoid rim fracture, is the mostly common seen intra-articular glenoid fracture type (type Ia, Ideberg-Goss classification system), approximately two-thirds of which are associated with shoulder dislocation.^2^

With improvements in arthroscopic techniques and the evolution of instruments over recent decades, arthroscopic management of bony Bankart lesions has shown similar function and recurrent instability outcomes compared with traditional open surgery, with a lower complication rate. Many arthroscopic techniques have been developed and modified for bony Bankart repair, including suture anchor, transosseous, and cannulated screw repairs.^3^, ^4^, ^5^, ^6^, ^7^, ^8^, ^9^, ^10^ In 2009, Millett and Braun^5^ reported the “bony Bankart bridge” technique involving an anchor on the articular and medial edges of the fracture with a suture bridge passed around the fragment by use of the shuttling technique with a 45° curved hook. The double-pulley suture-tying technique was originally reported by Arrigoni et al.^11^ and Koo et al.^12^ and applied in rotator cuff repair surgery and remplissage surgery. In 2011, Zhang and Jiang^6^ first applied this dual-row fixation (“double-pulley”) technique to arthroscopically restore and fix bony Bankart lesions, achieving satisfactory healing and biomechanical stability.

However, the arthroscopic procedures of “bony Bankart bridge” technique and double-pulley suture-tying technique for bony Bankart lesions can be very challenging technically. First, when trying to penetrate the soft tissues around the avulsed fracture fragment with the traditional suture device, it is not easy to manually control and adjust the suturing angle, and it may make the bony fragment and surrounding tissue split and cause iatrogenic injury. Second, in the reported technique, the suture device and grasper are difficult to operate simultaneously in the limited surgical space because of its larger diameter. Lastly, suture passing through the traditional portals makes suture management somewhat difficult and challenging.

In this Technical Note, we present a modified technique for arthroscopic reduction and double-pulley fixation of bony Bankart lesion by percutaneous spinal needle suture passing. The technique makes the arthroscopic treatment of bony Bankart lesion easier and more reproducible. Besides, it can provide stable, compressing, nontilting and reliable fixation for the fracture fragment. Our improved technique also does not require extra expensive consumables, making it more economically acceptable and applicable.

## Surgical Technique

The protocol of our technique is shown in Video 1. The main steps are described in the sections to follow.

### Step 1: Establishment of Different Observation Portals and Assessment of the Bony Bankart Lesion

**Fig 1:**
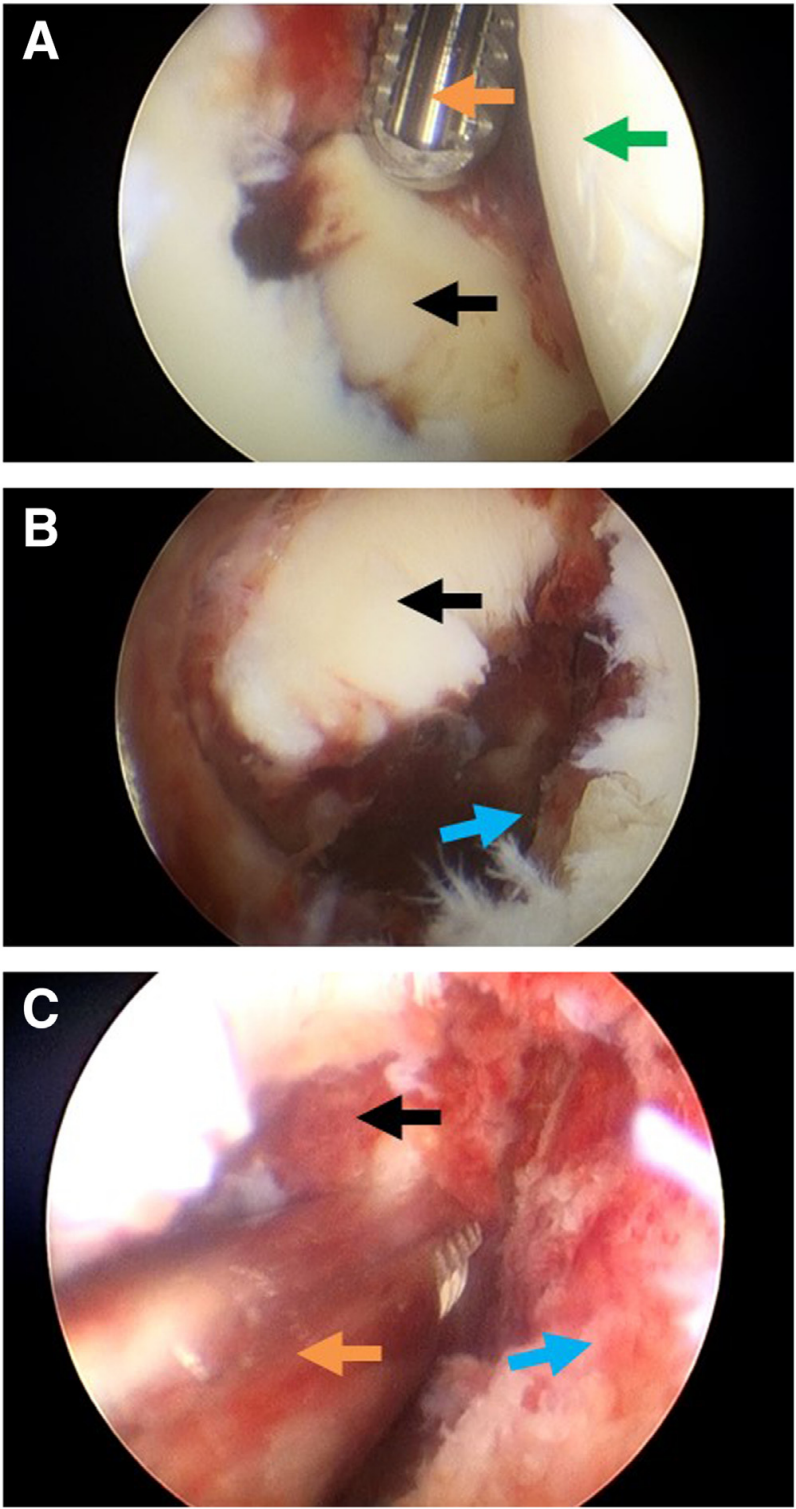
Establishment of different observation portals and assessment of the bony Bankart lesion. In this case, the female patient, 49 years old, has a right anterior glenoid rim fracture, with the right upper arm suspended at an abduction angle of 45° and anterior flexion of 15°, placed in a standard left lateral decubitus position throughout the entire operation. (A) Intra-articular view of a right glenohumeral joint from posterior viewing portal showing the bony Bankart lesion. (B) Intra-articular view of the bony Bankart lesion from the anterosuperior lateral viewing portal. (C) Cleaning of the hematoma, exposing the borderline of the fracture fragment. The black arrow indicates the Bankart fracture fragment. The green arrow indicates the humeral head. The orange arrow indicates the shaver. The blue arrow indicates the bony bed of Bankart lesion site.

General combined intravenous anesthesia and inhaled anesthesia is adopted during the arthroscopic operation. The patient is placed in a standard left lateral decubitus position with the right upper arm retracted by a 5-kg weight and suspended at an abduction angle of 45° and anterior flexion of 15°. After routine disinfection and draping, the standard posterior observation portal is created, and the anterior portal is created as operation channel. A 30° arthroscope is inserted through the standard posterior portal to observe the glenohumeral joint (Fig 1A). Once we confirm the presence of the bony Bankart lesion, the arthroscope is switched to the anterosuperior lateral portal to obtain a global view of the glenoid (Fig 1B) and to assess the severity of glenoid fracture. The hematoma is cleaned up by a shaver through the anterior operation portal to expose the borderline of the fracture fragment (Fig 1C).

### Step 2: Placement of the Medial Double-Loaded Suture Anchor and Procedures of Percutaneous Spinal Needle Suture Passing

**Fig 2:**
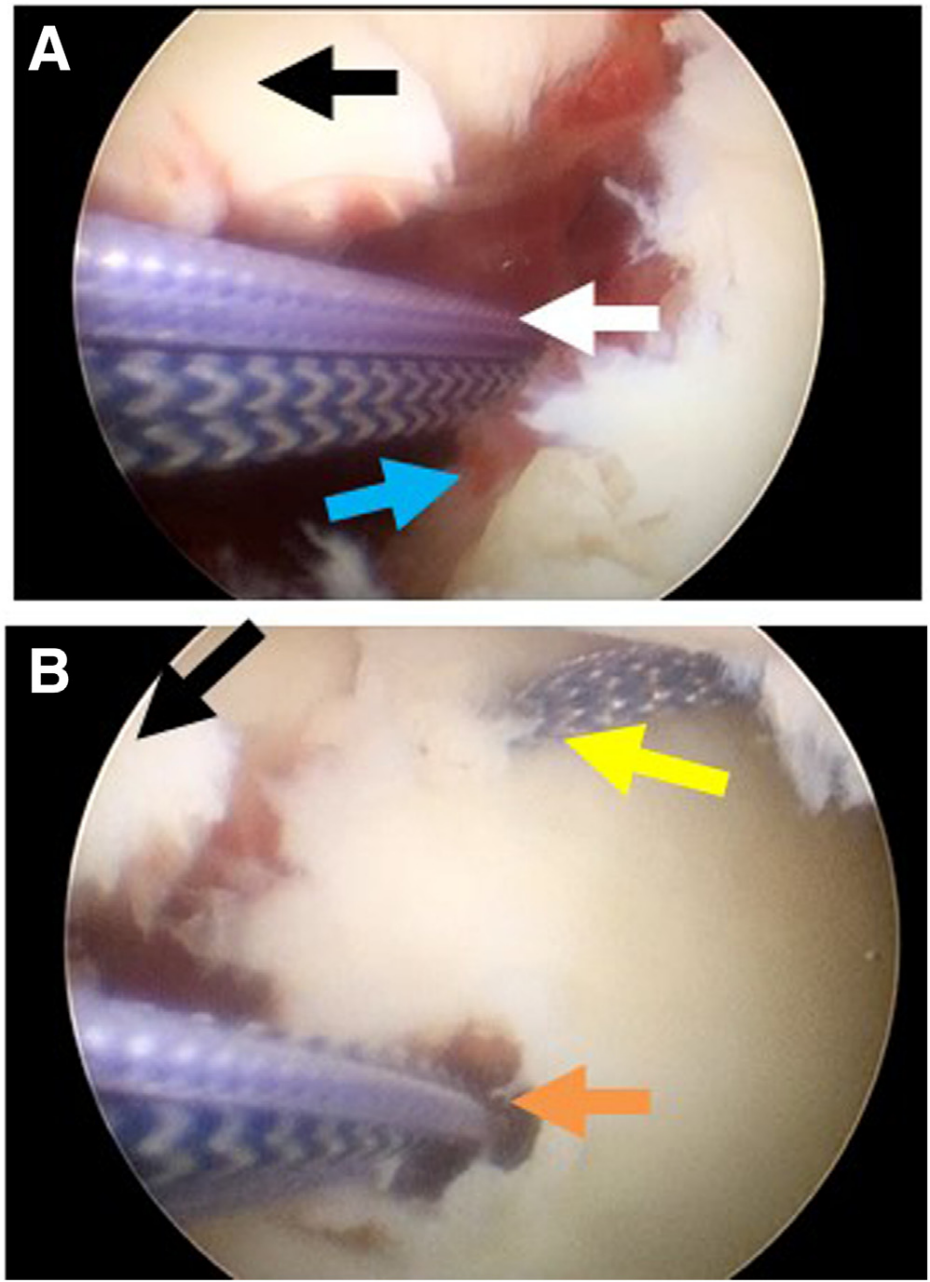
Placement of double-loaded suture anchors in the medial margin and lateral articular edge of the bony bed of Bankart lesion site. The anterosuperior lateral portal is used as the observation portal, the anterior portal is used as the operating portal. (A) A double-loaded suture anchor (as indicated by the white arrow) is positioned in the midpoint of medial margin of the bony bed. (B) Two double-loaded suture anchors are respectively placed proximally (as indicated by the orange arrow) and distally (as indicated by the yellow arrow) in the lateral articular edge of the bony bed of Bankart lesion site. The black arrow indicates the Bankart fracture fragment. The blue arrow indicates the bony bed of Bankart lesion site.

**Fig 3:**
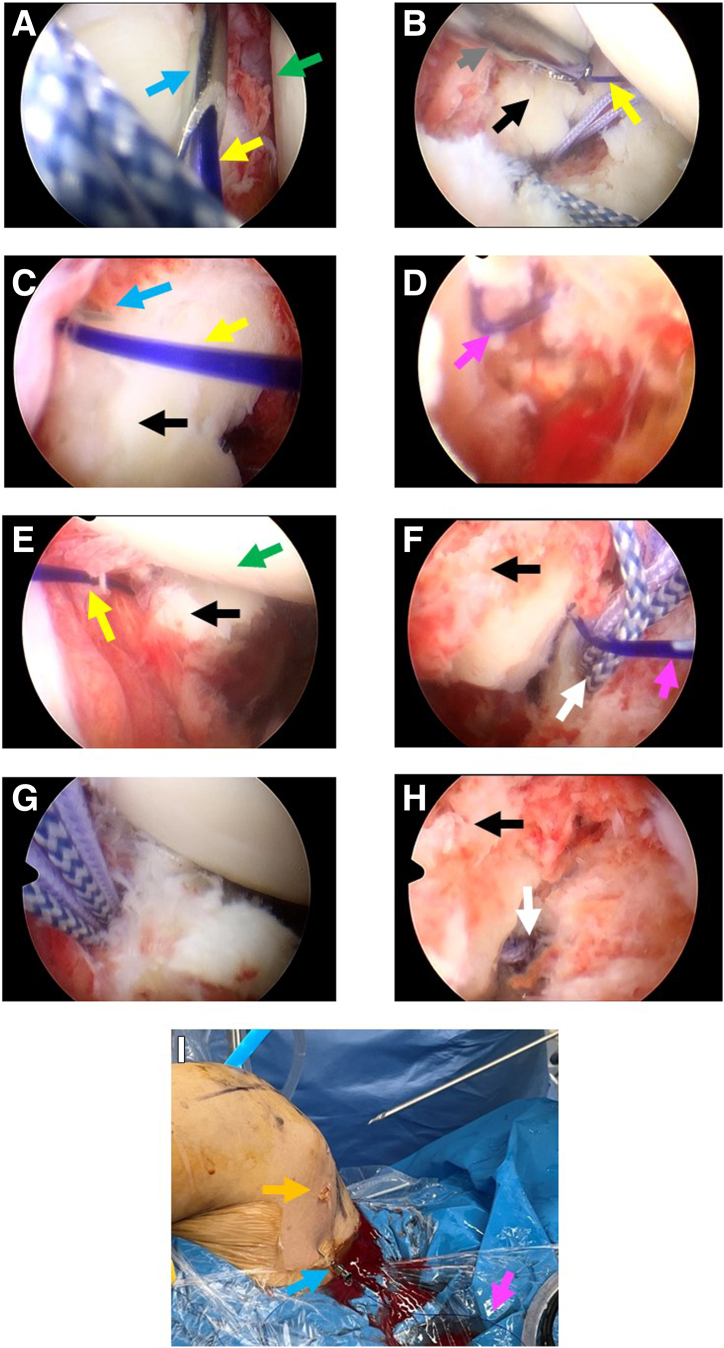
Procedures of spinal needle with PDS suture percutaneously penetrating to the medial and articular edge of fracture fragment, and suture passing. The posterior portal is used as the observation portal. (A) A 12-gauge spinal needle is inserted percutaneously below the anterior portal, penetrating through the subscapularis tendon and capsule, reaching the midpoint of the articular surface of the glenoid fracture fragment. One end of a PDS suture is introduced into the joint through the spinal needle. The anterosuperior lateral portal is used as the observation portal, (B) The end of PDS suture inside the joint is pulled out using a suture grasper through the anterior approach. (C) Retrieving the spinal needle back outside the joint capsule. (D) The spinal needle is inserted again along the front beveled edge of the Bankart fracture fragment, penetrating the capsule and reaching below the Bankart fracture fragment. Exposing the tip of the spinal needle with PDS suture. (E) PDS suture strand in the articular edge of the glenoid fracture fragment. (F) The other PDS suture strand below the Bankart fracture fragment. (G) The suture strands of the anchor placed in the medial margin of bony bed are pulled out with the high tensile traction suture. (H) The anchor sutures are passed through the medial edge of the fracture fragment. (I) Extra-articular view. In this figure, the black arrow indicates the Bankart fracture fragment. The blue arrow indicates the spinal needle. The yellow arrow indicates the PDS suture strand passing the articular edge of the glenoid fracture fragment. The pink arrow indicates the other PDS suture strand passing the medial edge of the glenoid fracture fragment. The green arrow indicates the humeral head. The white arrow indicates the sutures of the anchor placing in the medial margin of the bony bed of Bankart lesion. (PDS, polydioxanone.)

**Fig 4:**
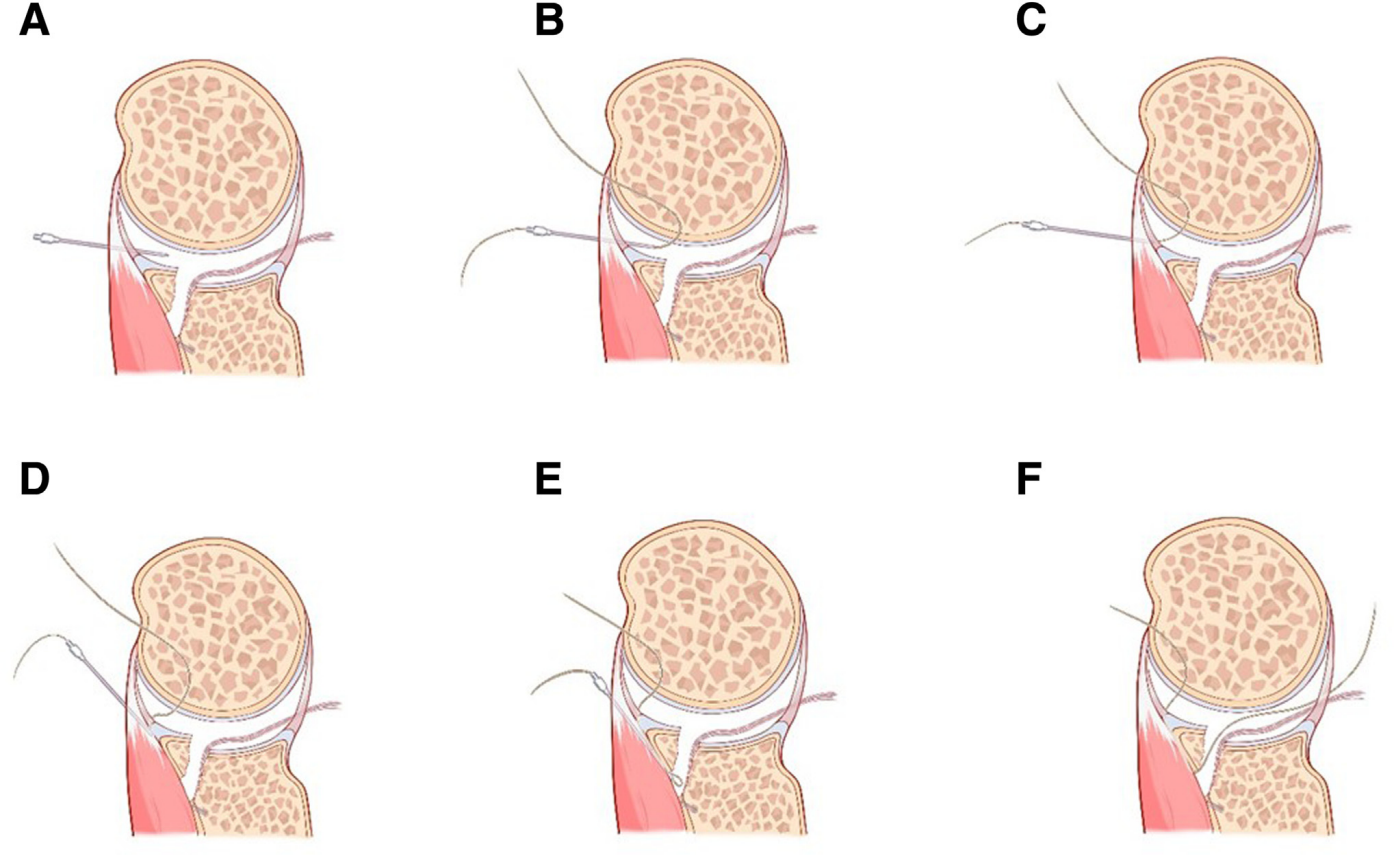
From the cross-sectional view, schematic diagram of the procedures of percutaneous spinal needle suture passing. (A) Insertion of the 12-gauge spinal needle percutaneously below the anterior portal to penetrate the subscapularis tendon and capsule, reaching the midpoint of the articular surface of the glenoid fracture fragment. (B) Introduction of one PDS suture end into the glenohumeral joint through the spinal needle. The arthroscope is switched to the anterosuperior lateral portal, and the end of PDS inside the joint is pulled out using a suture grasper through the anterior approach. (C-E) Retrieving the spinal needle back outside the joint capsule, and then insert the spinal needle again along the front beveled edge of the avulsed fracture fragment, penetrating the capsule and reaching below the Bankart fracture fragment. (F) Pulling out the other end of the PDS by a suture grasper through the posterior approach. (PDS, polydioxanone.)

The anterosuperior lateral portal is used as the observation portal, through the anterior operating portal, a double-loaded suture anchor (LUPINETM BR Anchor W/DS ORTHOCORDTM; DePuy Mitek) is implanted in the midpoint of medial margin of the bony bed as the medial row (Fig 2A). The anchor sutures then are pulled out with a suture grasper through the posterior approach. With the standard posterior observation portal, we insert the 12-gauge spinal needle percutaneously below the anterior portal to penetrate subscapularis tendon and capsule, reaching the midpoint of the articular surface of the glenoid fracture fragment (Fig 3A). One end of a polydioxanone (PDS) suture (PDS II; Ethicon, Somerville, NJ) is introduced into the glenohumeral joint through the spinal needle. The arthroscope is switched to the anterosuperior lateral portal, and the end of PDS inside the glenohumeral joint is pulled out using a suture grasper through the anterior approach (Fig 3B). The spinal needle is retrieved back outside the joint capsule (Fig 3C), and then it is inserted the spinal needle again along the front beveled edge of the avulsed fracture fragment, penetrating the capsule and reaching medial midpoint of the Bankart fracture fragment (Fig 3D). The second penetration is a blind process; sometimes it is not easy to find the needle tip and PDS suture. Thus, it is highly necessary to perform this step strictly along the edge and bevel of the fracture fragment when penetrating. The suture grasper can be exploited to expand the fracture gap, assisting in exposing the spinal needle tip, finding and catching the other PDS suture end. The caught PDS suture end is then pulled by a suture grasper through the posterior approach. This moment, the PDS suture strands are passed through the soft tissues medial to the bony fracture fragment (Fig 3 E and F). Figure 4 schematically illustrates the steps of percutaneous spinal needle suture passing from the cross-sectional view. Outside the glenohumeral joint, the PDS suture is replaced with a high-tensile suture (Orthocord Suture; #2 Violet W/ MO-7 1/2 Circle, Taper Point Needle, 22 mm; DePuy Mitek), which functions as a traction suture. From the posterior approach, all 4 limbs of the medial-row anchor suture strands are passed through the medial edge of the fracture fragment with the high tensile traction suture. All suture strand limbs are pulled out through the anterior approach by a suture grasper (Fig 3 G and H). Figure 3I provides an extra-articular view of this process.

### Step 3: Placement of the Lateral Double-Loaded Suture Anchors and Fixation and Stabilization of the Fracture Fragment by Double-Pulley Technique

**Fig 5:**
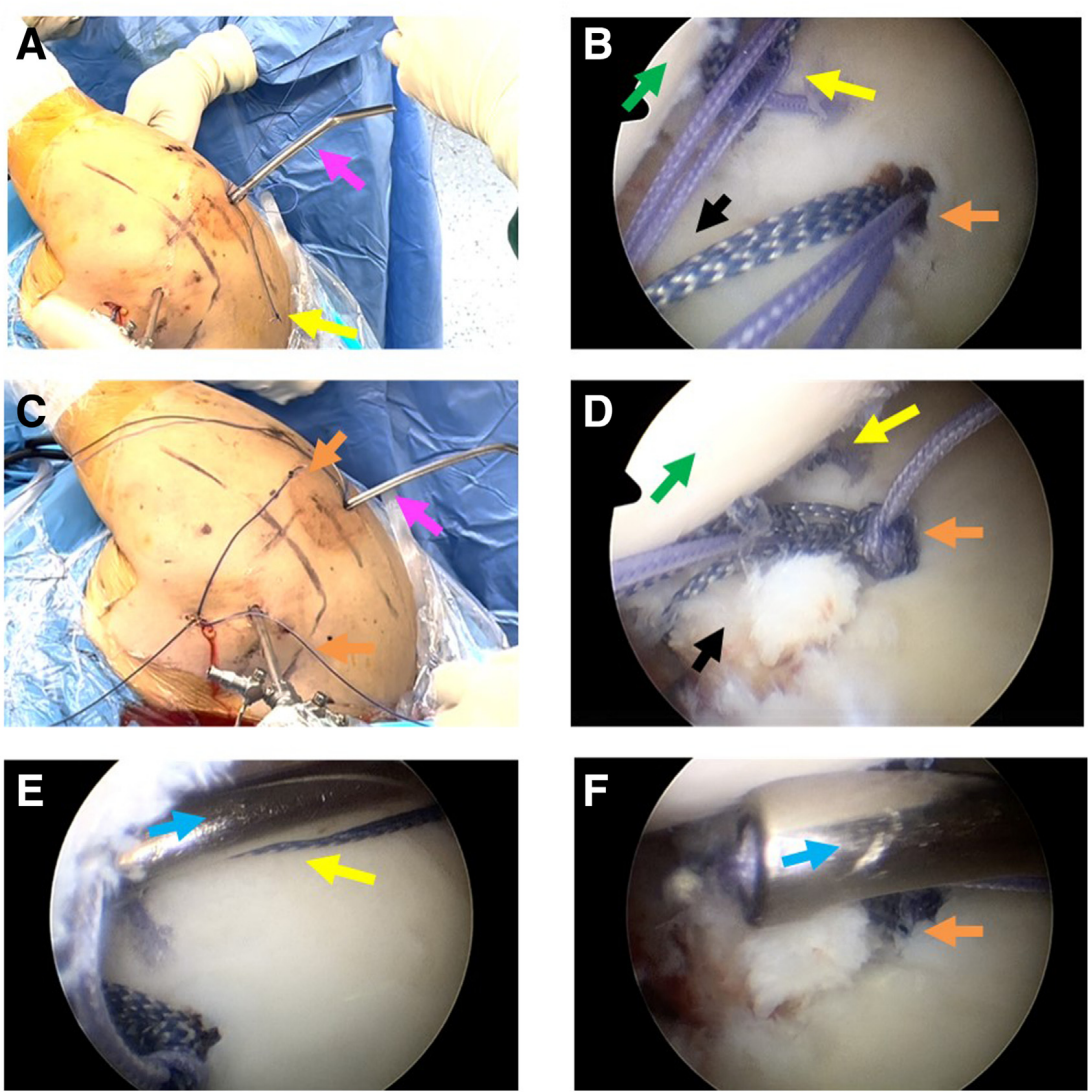
Fixation and stabilization of the fracture fragment by double-pulley technique. The anterosuperior lateral portal is used as the observation portal. (A) A semi-open retractor is placed as operating channel through posterior portal. Strands of one suture from the articular distal anchor and strands of one suture from the medial anchor are pulled out. Two strands from different sutures are tied outside of the joint with a static surgeon’s knot. (B) Delivering the knot through the channel and down back to the fracture site. (C) The sutures of the medial anchor and articular proximal anchor are manipulated as the same procedure through the anterior approach. (D) Delivering the knot back to fracture site. (E-F) The 2 left free strands of different sutures are retrieved, and a static knot is tied with the knot pusher through the posterior portal, finally the fracture fragment is fixed and stabilized by double-pulley technique. In this figure, the black arrow indicates the Bankart fracture fragment. The blue arrow indicates the knot pusher. The pink arrow indicates the semi-open retractor. The green arrow indicates the humeral head. The yellow arrow indicates the 2 strands of different sutures from the articular distal anchor and the medial anchor. The orange arrow indicates the 2 strands of different sutures from the articular proximal anchor and the medial anchor.

**Fig 6:**
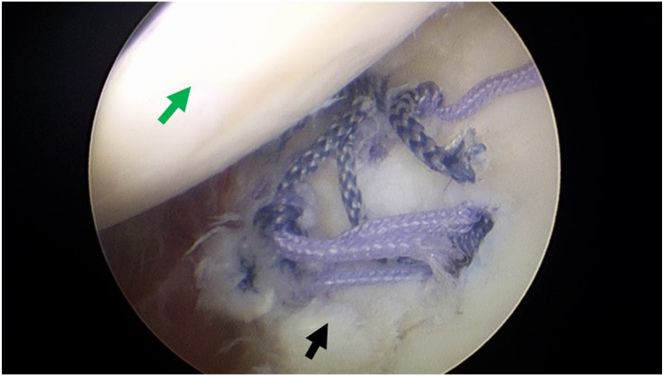
Exploration and assessment of the fracture fragment after double-pulley technique fixation. The anterosuperior lateral portal is used as the observation portal. The final construct of double-pulley technique fixation comprises 8 suture strands and 4 knots overlying the fracture site in 2 double mattress configurations and there are in total 4 loops running across the fracture fragment horizontally, firmly compressing the fracture fragment against the glenoid bone bed. In this figure, the black arrow indicates the stabilized Bankart fracture fragment. The green arrow indicates the humeral head.

**Fig 7:**
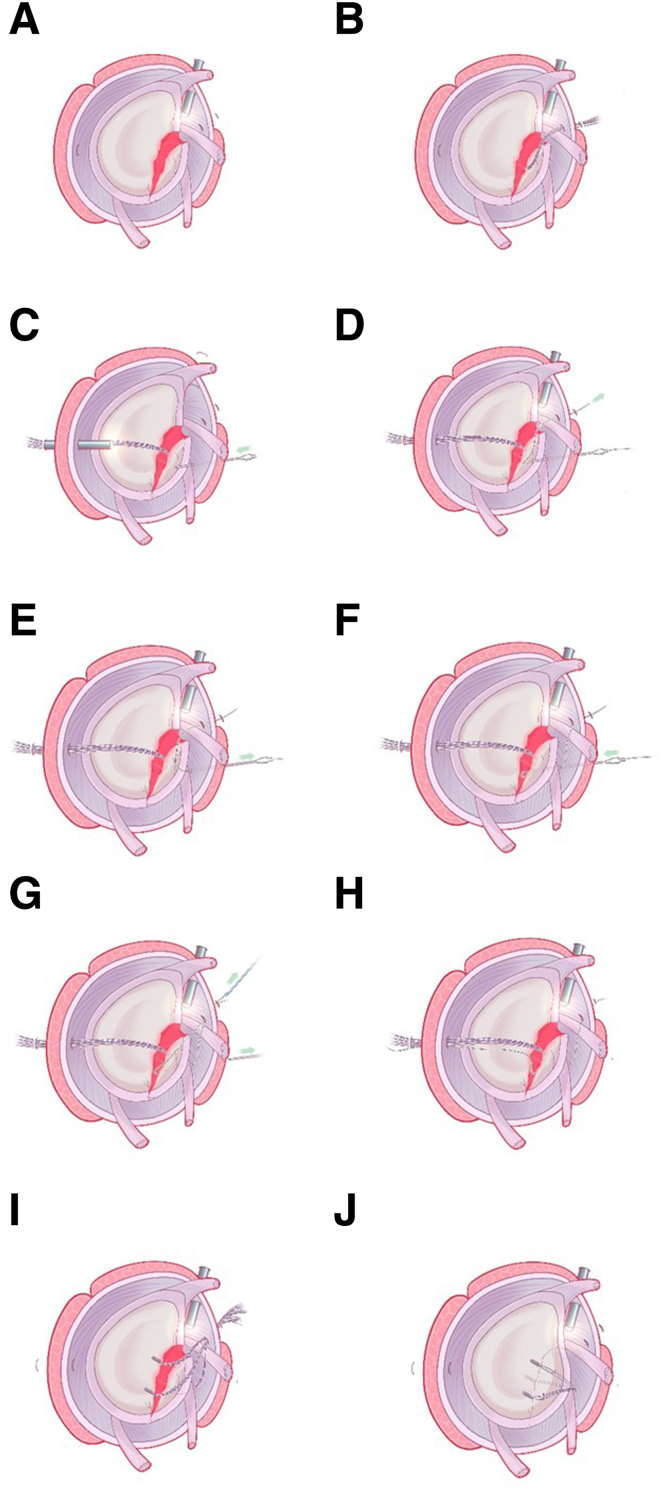
From the oblique sagittal view, schematic diagram of our technique for arthroscopic reduction and double-pulley fixation of bony Bankart lesion by percutaneous spinal needle suture passing. (A) Establishment of anterosuperior lateral portal and assessment of the bony Bankart lesion. (B) With the anterosuperior lateral observation portal and the anterior operation portal, placement of the medial-row anchor in the midpoint of medial margin of the bony bed. (C) With the standard posterior observation portal, insertion of the 12-gauge spinal needle percutaneously below the anterior portal to penetrate the subscapularis tendon and capsule, reaching the midpoint of the articular surface of the glenoid fracture fragment. Introduction of one PDS suture end into the glenohumeral joint through the spinal needle. (D) The arthroscope is switched to the anterosuperior lateral portal, and the end of PDS inside the joint is pulled out using a suture grasper through the anterior approach. (E-H) Retrieving the spinal needle back outside the joint capsule, and then inserting the spinal needle again along the front beveled edge of the avulsed fracture fragment, penetrating the capsule and reaching medial midpoint of the Bankart fracture fragment, and pulling out the second PDS suture end by a suture grasper through the posterior approach. (I) Placement of the lateral doubly loaded suture anchors through the anterior operation portal. (J) Fixation and stabilization of the fracture fragment by double-pulley technique. (PDS, polydioxanone.)

**Fig 8:**
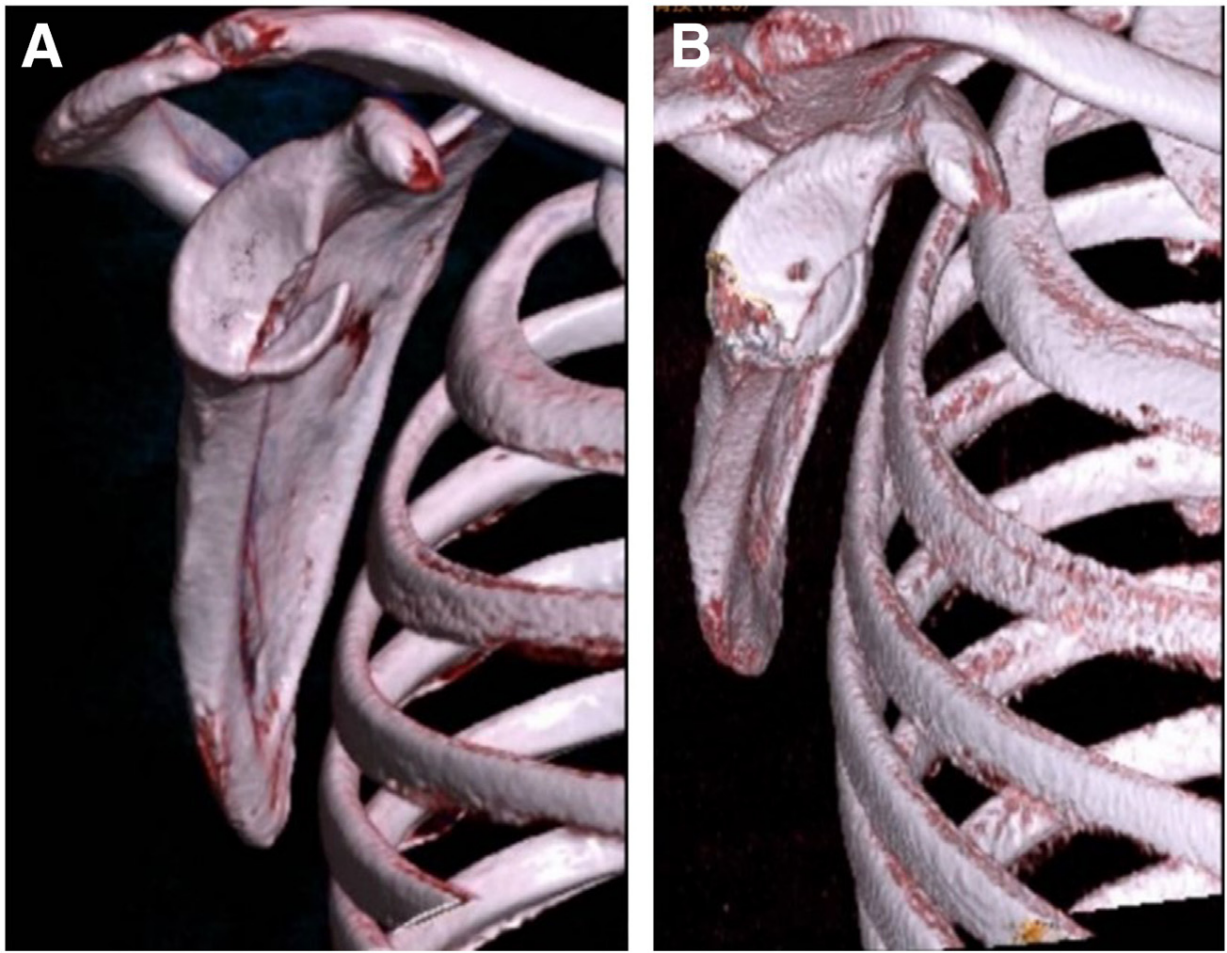
Preoperative and postoperative images of the bony Bankart lesion. (A) Preoperative images of the bony Bankart lesion on 3D CT reconstruction of the “en face view.” (B) Postoperative images of the bony Bankart lesion on 3D CT reconstruction of the “en face view,” showing anatomical reduction and stable fixation of the fragment. (3D, 3-dimensional; CT, computed tomography.)

The anterosuperior lateral portal is used as the observation portal, and the anterior portal is used as operation portal, 2 doubly loaded suture anchors (LUPINETM BR Anchor W/DS ORTHOCORDTM; DePuy Mitek) are respectively positioned proximally one-third and distally one-third in the lateral articular edge of the bony bed as the lateral row (Fig 2B). Suture strands of the distal anchor are pulled out through the posterior approach. A semiopen retractor is placed as operating channel through posterior portal. Strands of one suture from the articular distal anchor and strands of one suture from the medial anchor are pulled out. Two strands from different sutures are tied outside of the glenohumeral joint with a static surgeon’s knot (Fig 5A). The other 2 free strands are pulled and tightened; this delivers the knot through the channel and down back to the lesion site (Fig 5B). The sutures of the medial anchor and articular proximal anchor are manipulated as the same procedure through the anterior portal (Fig 5C). Delivering the knot back fracture site (Fig 5D), then the 2 left free strands of different sutures are retrieved, and a static knot is tied with the knot pusher through the posterior portal, stabilizing the fracture fragment by double-pulley technique (Fig 5E and F). The left unknotted strands loaded on lateral anchors are pulled out and abandoned. The final construct comprises 8 suture strands and 4 knots overlying the fracture site in 2 double mattress configurations and there are in total 4 loops running across the fracture fragment horizontally, firmly compressing the fracture fragment against the glenoid bone bed (Fig 6). Figure 7 shows the schematic diagram of the entire technique for arthroscopic reduction and double-pulley fixation of bony Bankart lesion by percutaneous spinal needle suture passing from the oblique sagittal view. Depending on the size of the fracture fragment, a strategy of 3 or 5 anchors can be adopted and technically placed by the surgeon after arthroscopic assessment. Shown as 3-dimensional computed tomography reconstruction images, an arthroscopic osteosynthesis was finally achieved (Fig 8A and B).

## Discussion

Fracture of the anterior glenoid rim, also called as bony Bankart lesion, is highly associated with anterior glenohumeral instability. Conservative therapy is recommended to patients with small, concentric reduced bony Bankart lesions (<5%); however, in cases of chronic Bankart fractures or bone loss with fragments that cannot be reduced or when there has been resorption of the bone fragment, surgeons consider other techniques, such as an open or arthroscopic Latarjet procedure or iliac crest bone graft reconstruction, into account.^1^^,^^13^ When radiographic and arthroscopic assessment shows a ununited Bankart fracture fragment attached to the glenoid bone bed, an arthroscopically osseous repair and fixation is recommended, the technique described in this article can be exploited to easily, conveniently, and economically restore and fix fracture fragment arthroscopically.

Although many wonderful arthroscopic techniques that reduce and fix bony Bankart lesion have been reported, the key and challenging step of reported arthroscopic bony Bankart surgery is suture passing, especially for large Bankart bony fragments. In using the shuttling technique in reported articles with traditional suture devices, such as curved suture hook, suture passers, and penetrators, surgeons may encounter several problems during suture-passing process, including splitting the fracture fragment or surrounding soft tissue, resulting in iatrogenic damage, creating difficulties for surgical apparatus operation and suture management. Thus, this places stringent requirements on the experience and skills of the surgeons. Our technique solves this problem with a 12-gauge spinal needle. It has the following potential advantages during the challenging suture passing step: Theoretically, the ideal suture tool should have the smallest possible diameter; a spinal needle is much smaller in diameter compared with traditional suture devices. It will allow for minimal iatrogenic injuries, and penetration can be attempted and performed multiple times but will not split the fracture fragment or surrounding soft tissue. Second, percutaneous spinal needle penetration has lower requirements for suturing angles, and straight penetrating operation is easier to manually control and adjust the suturing angle or insertion point compared with traditional suture devices. It is more suitable for beginners with a learning curve. Third, percutaneous spinal needle suture passing with an additional invisible working channel will avoid interference between the suture device and grasper, save space for other arthroscopic instruments, and make suture management easier, significantly reducing the operational difficulties for the surgeon. Lastly, our technique is strategically applicable to Bankart fracture fragments of different sizes, shapes, and locations. Our suture passing technique can be combined with other anchor-dependent arthroscopic techniques to fix bony Bankart lesion, but not just double pulley fixation technique.

There are some concerns regarding this modified technique for arthroscopic reduction and double-pulley fixation of bony Bankart lesion. First, when the insertion site or direction is incorrect, there is a risk of causing damage to the tissue passing through, including vessels and nerves tissue. Therefore, percutaneous spinal needle penetration requires additional learning and training, it has a learning curve. Second, the double-pulley fixation in this patient comprises 8 suture strands and 4 knots overlying the fracture site, suture ends cannot be cut completely flush with the articular cartilage. Those knots and loose ends of the sutures may cause wear and tear and damage to glenohumeral cartilage during shoulder motion.

In this Technical Note, we have mentioned a simplified and reproducible technique for arthroscopic reduction and double-pulley fixation of bony Bankart lesions by percutaneous spinal needle suture passing, which is inspired by our previous research on the arthroscopic subscapularis repair by percutaneous spinal needle suture passing.^14^ Compared with the reported technique for arthroscopic reduction and fixation of bony Bankart lesions by traditional suture devices, our technique is more minimal invasive to patients, is more convenient and economical, and is more suitable for beginners to learn. It is a stable way of arthroscopically treating bony Bankart lesions. In addition, the equipment used in our technique is commonly-seen and accessible in the operating room, without requiring high-value consumables and supporting instruments. This increases the cost-effectiveness and feasibility of this surgical technique. We summarize and list the pearls and pitfalls of our technique in Table 1 and the advantages and disadvantages in Table 2. In summary, our modified technique has a very good application value in arthroscopic reduction and fixation of the bony Bankart lesion, which integrates the advantages of economy, simplicity, reliability and safety.

**Table 1:** The Pearls and Pitfalls of Our Technique for Arthroscopic Reduction and Double-Pulley Fixation of Bony Bankart Lesion by Percutaneous Spinal Needle Suture Passing

Pearls	Pitfalls
1. The anterosuperior lateral portal is the main observation portal and the posterior portal can be used as an accessory observation portal, especially switching the arthroscope to posterior portal may be more suitable for operator's habits during the percutaneous spinal needle penetration.	1. The second time percutaneous spinal needle penetration is a blind process, sometimes it is not easy to find the needle tip and PDS suture. Thus, it is necessary to strictly along the front beveled edge of the fracture fragment when penetrating. The grasper can be exploited to expand the fracture gap, assisting in exposing the surgical field.
2. When performing percutaneous spinal needle penetration, the ideal penetrating direction is parallel to the glenoid articular surface, and the ideal penetrating destination for first time is the midpoint of the articular surface of the glenoid fracture fragment.	2. When performing percutaneous spinal needle penetration, the skin insertion point should not be too low or inward to avoid damaging vessels and nerves.
3. Before the second time penetration, spinal needle should be retrieved back outside the joint capsule, and then insert the spinal needle again along the front beveled edge of the avulsed fracture fragment, backwardly penetrating the capsule and reaching below the Bankart fracture fragment. The second time penetration is a blind process, and the main reference mark is the front beveled edge of the fracture fragment. Then find and catch the end of PDS suture by a suture grasper through the anterior approach.	
4. In case of the breakage of PDS suture during traction, it is highly necessary to replace the PDS suture with a high-tensile suture, then tract and pull out all 4 strands of medial anchor sutures with high-tensile suture.	
5. The dual-row fixation (“double-pulley”) technique is applied to restore and fix fracture fragment in this technique. Three or five anchors can be technically placed depending on the size of the fracture fragment.	

PDS, polydioxanone.

**Table 2:** Advantages and Disadvantages of Our Technique for Arthroscopic Reduction and Double-Pulley Fixation of Bony Bankart Lesion by Percutaneous Spinal Needle Suture Passing

Advantages	Disadvantages
1. The key and challenging step of arthroscopic bony Bankart surgery is suture passing, especially for large Bankart bony fragments, the shuttling technique with a curved suture hook has the possibility of splitting the fracture fragment and surrounding tissue when penetrating surrounding soft tissues. Our technique solves this problem and makes the challenging suture passing step easier.	1. Percutaneous spinal needle penetration has a learning curve, it requires additional learning and training.
2. Spinal needle is smaller compared with suture hook, which will reduce iatrogenic injuries. It can be tried and performed for multiple times and has greater error tolerance.	2. Percutaneous spinal needle penetration may cause damage to the tissue passing through.
3. Percutaneous spinal needle penetration has lower requirement for suturing angles, additionally straight penetrating operation is easier compared with curved suture hook.	
4. Percutaneous spinal needle penetration passing makes suture management easier with an additional invisible working channel.	
5. Our technique is strategically applicable to Bankart fracture fragments of different sizes, shapes, and locations. 6. Our suture passing technique can be combined with other anchor-dependent arthroscopic techniques to fix bony Bankart lesion, but not just double pulley fixation technique.	

## Disclosures

All authors (F.W., W.Y., C.M., H.W., W.H.) declare that they have no known competing financial interests or personal relationships that could have appeared to influence the work reported in this paper.

## Supplementary Data

Video 1 Introduction of the simplified technique for arthroscopic reduction and double pulley fixation of bony Bankart lesion by percutaneous spinal needle suture passing. The key steps of the technique are illustrated. ① Female patient, 49 years old, with the right upper arm suspended at an abduction angle of 45° and anterior flexion of 15°, is placed in a standard left lateral decubitus position. The glenohumeral arthroscopy is performed through the standard observation posterior portal and the anterior approach serves as the operating portal. The hematoma is cleaned up by a shaver, confirming the presence of the bony Bankart lesion. ② The arthroscope is switched to the anterosuperior lateral portal, where a global view of the glenoid can be obtained. Cleaning up the hematoma in the lesion site by a shaver, till to the medial edge of the glenoid bony Bankart lesion site. ③ Through the anterior operating portal, a doubly-loaded suture anchor is strategically inserted into the midpoint of the medial margin of the glenoid bone bed, where the bony Bankart lesion occurred. Pulling out the anchor sutures by a suture grasper through the posterior approach. ④ With the standard observation posterior portal, we insert the 12-gauge spinal needle percutaneously below the anterior portal to penetrate the subscapularis tendon and capsule, reaching the midpoint of the articular surface of the glenoid fracture fragment. One end of a PDS suture is introduced into the glenohumeral joint through the spinal needle. The arthroscope is switched to the anterosuperior lateral portal, and the end of PDS inside the joint is pulled out using a suture grasper through the anterior approach. Retrieving the spinal needle back outside the joint capsule, and then insert the spinal needle again along the front bevelled edge of the avulsed fracture fragment, penetrating the capsule and reaching below the Bankart fracture fragment. Pulling out the other end of the PDS by a suture grasper through the posterior approach. ⑤ Outside the glenohumeral joint, replacing the PDS suture with a high-tensile suture, which functions as a traction suture. From the posterior approach, all 4 limbs of the medial anchor suture strands are passed through the medial edge of the fracture fragment with the traction suture. Then pull out all suture strand limbs through the anterior approach. With the observation anterosuperior lateral portal, and the operation anterior portal. ⑥ Respectively, 2 doubly loaded suture anchors are positioned proximally one-third and distally one-third of the lateral articular edge of the glenoid bone bed. ⑦ A semi-open retractor is placed as operating channel through posterior portal. Strands of one suture from the articular distal anchor and strands of one suture from the medial anchor are pulled out. Two strands from different sutures are tied outside of the joint with a static knot, the other 2 free strands of different sutures are pulled and tightened, this delivers the knot through the channel and down back to the lesion site. The sutures of the medial anchor and articular proximal anchor are manipulated as the same procedure through the anterior portal. Delivering the knot back fracture site, then the left 2 free strands of different sutures are retrieved, and a static knot is tied with the knot pusher. An arthroscopic osteosynthesis is satisfactorily achieved.
